# *Ctdp1* deficiency leads to early embryonic lethality in mice and defects in cell cycle progression in MEFs

**DOI:** 10.1242/bio.057232

**Published:** 2021-01-06

**Authors:** Fangfang Qiao, Henry C.-H. Law, Kimiko L. Krieger, Emalie J. Clement, Yi Xiao, Shannon M. Buckley, Nicholas T. Woods

**Affiliations:** 1 Eppley Institute for Research in Cancer and Allied Diseases, Fred & Pamela Buffet Cancer Center, University of Nebraska Medical Center, Omaha, NE 68198, USA; 2 Department of Genetics, Cell Biology, and Anatomy, University of Nebraska Medical Center, Omaha, NE 68198, USA

**Keywords:** Ctdp1, Knockout, CCFDN, Embryonic lethality, MEFs, Cell death, Cell cycle arrest

## Abstract

RNA polymerase II subunit A Carboxy-Terminal Domain Phosphatase 1 (CTDP1), a member of the haloacid dehalogenase superfamily phosphatases, has a defined role in transcriptional regulation, but emerging evidence suggests an expanded functional repertoire in the cell cycle and DNA damage response. In humans, a splice site mutation in *CTDP1* gives rise to the rare Congenital Cataracts Facial Dysmorphism and Neuropathy syndrome, and recent evidence from our lab indicates CTDP1 is required for breast cancer growth and proliferation. To explore the physiological function of CTDP1 in a mammalian system, we generated a conditional *Ctdp1* knockout mouse model by insertion of *loxP* sites upstream of exon 3 and downstream of exon 4. Biallelic deletion of *Ctdp1* results in lethality before embryonic day 7.5, with morphological features indicating embryo cell death and resorption. However, *Ctdp1*^+/−^ mice are haplosufficient for phenotypic traits including body weight, hematological parameters, exploratory and locomotive functions. To investigate the potential mechanisms of the embryonic death caused by biallelic *Ctdp1* knockout, mouse embryonic fibroblasts (MEFs) were established from *Ctdp1*^+/+^ and *Ctdp1*^flox/flox^ mice. Lentivirus delivered Cre-mediated biallelic deletion of *Ctdp1* in MEFs results in cell death preceded by impaired proliferation characterized by an increase in G1- and G2-phase populations and a reduction in the S-phase population. These cell cycle alterations caused by deletion of *Ctdp1* are associated with an increase in p27 protein expression and a decrease in phosphorylated RB, phosphorylated Histone H3, and Cyclin B expression. Together, these results reveal that *Ctdp1* plays an essential role in early mouse embryo development and cell growth and survival in part by regulating the cell cycle.

## Abstract (precis)

**Summary:** Knockout of *Ctdp1* reveals its essential role in mammalian embryogenesis and regulation of the cell cycle.

## INTRODUCTION

CTDP1 dephosphorylates the C-terminal domain (CTD) of the RNA polymerase II subunit RPB1, preparing the complex for repeated rounds of transcription initiation. CTDP1 interacts with the C-terminal domain of the RAP74 subunit of transcription initiation factor TFIIF and its phosphatase activity is dependent on the stimulation of TFIIF, thus it is also known as TFIIF-associating component of CTD phosphatase (FCP1). CTDP1 contains an FCP1 phosphatase homology domain and a BRCA1 C-terminal (BRCT) domain, which binds and dephosphorylates the CTD of RPB1 (Yu et al., 2003; Glover et al., 2004). The singleton BRCT domain in CTDP1 is also essential for its function in transcription (Kobor et al., 2000; Majello and Napolitano, 2001). Recent studies have also revealed novel transcription-independent functions of CTDP1, including regulation of mitosis and the DNA damage response (DDR) (Son and Osmani, 2009; Visconti et al., 2012; Jeong et al., 2005; Hu et al., 2019).

In human disease, Congenital Cataracts Facial Dysmorphism and Neuropathy (CCFDN) is characterized by delayed early motor and intellectual development. CCFDN is caused by the splicing variant IVS6+389C>T in intron 6 of *CTDP1*, leading to the production of 30% functional CTDP1 protein and 70% truncated protein (Varon et al., 2003; Kalaydjieva, 2006). In addition, there is emerging evidence of an association between CTDP1 and cancer (Fu et al., 2015; Zhong et al., 2016). Indeed, a recent study by our lab found that CTDP1 knockdown not only increases the sensitivity of breast cell lines to DNA inter-strand crosslink (ICL)-inducing agents but also compromises the growth of breast cancer cells *in vitro* and *in vivo* (Hu et al., 2019). These results suggest CTDP1 is an important regulator of cellular pathways and processes implicated in human disease.

Previous studies using *Drosophila* have demonstrated that *Fcp1* is indispensable for normal developmental processes (Tombácz et al., 2009; Juhász et al., 2012). Thus far, however, the developmental and disease-associated role of CTDP1 has not been examined in vertebrate systems. To study the physiological functions of mammalian *Ctdp1*, we targeted this gene and created a conditional knockout (KO) mouse model. In the initial characterization of this mouse, we aimed to investigate the role of *Ctdp1* in mouse embryonic development and whether the heterozygous mice could display CCFDN-like phenotypes. Loss of both *Ctdp1* alleles results in lethality prior to embryonic day 7.5 (E7.5) of gestation. Unexpectedly, partial loss of *Ctdp1* in heterozygous mice does not elicit a CCFDN-like phenotype. We examined the function of *Ctdp1* in the growth of mouse embryonic fibroblasts (MEFs) and determined the loss of cell viability is associated with alternations in cell cycle progression and expression of cell cycle regulators. Together, these results indicate *Ctdp1* is an essential gene during early mammalian embryo developmental processes and provide additional evidence that *Ctdp1* regulates cell cycle progression.

## RESULTS AND DISCUSSION

### Generation of Ctdp1 KO mice

**Fig. 1.**
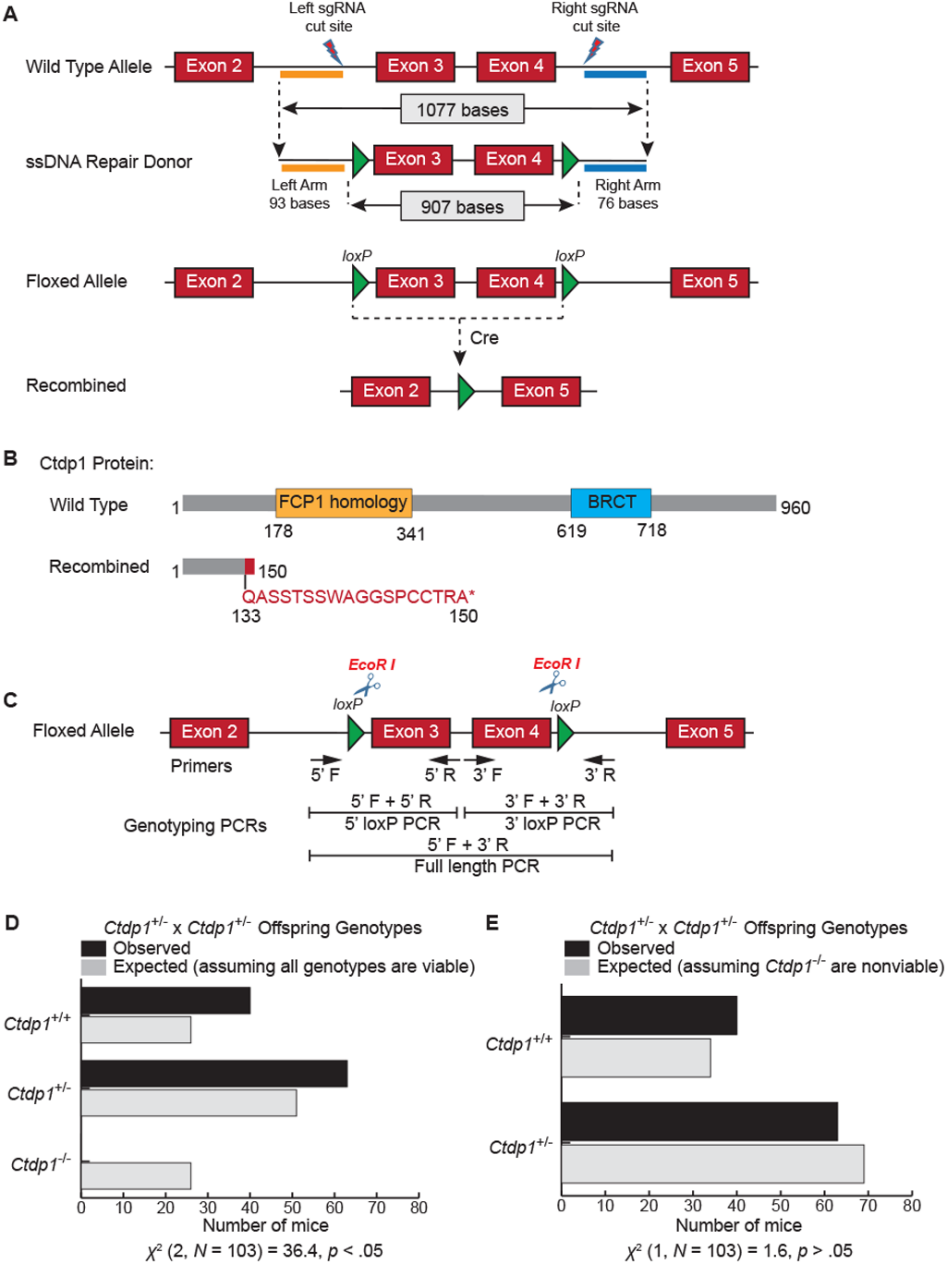
**Generation of the conditional *Ctdp1* KO mice.** (A) Schematic representation of the mouse *Ctdp1* wild-type allele, the *Ctdp1* ssDNA repair donor construct, and the predicted recombined product. (B) The modular domain organization of the mouse Ctdp1 protein (top) and the product after deletion of the floxed alleles (bottom). (C) The three genotyping PCR strategies used in this study. (D,E) Bar charts representing the expected and observed number of mice with the indicated genotypes from *Ctdp1*^+/−^ intercrosses assuming that the *Ctdp1*^+/−^ genotype is either viable (D) or nonviable (E). The chi-squared test of independence result is presented below the charts.

We generated a *Ctdp1* KO mouse model using the Cre-*loxP* system to explore the *in vivo* physiological functions of this gene. Exons 3 and 4 of the *Ctdp1* gene were targeted as the KO region and flanked by two *loxP* sequences oriented in the same direction using the strategy depicted in Fig. 1A. After the expression of Cre, the deletion of exons 3 and 4 causes a frameshift that results in the addition of 13 out of frame amino acids and a stop codon (Fig. 1B). The *Ctdp1^flox/flox^* mice were viable, born at the expected frequency, fertile and did not exhibit distinguishable phenotypes compared with their littermate controls. To obtain heterozygous *Ctdp1^+/−^* mice, we crossed *Ctdp1^flox/flox^* mice with E2a-Cre transgenic mice that express Cre recombinase under the control of the E2a promoter. The E2a-Cre system triggers Cre expression in early mouse embryo tissues including the germ cells. The *Ctdp1^+/−^* mice were intercrossed in an attempt to generate homozygous *Ctdp1^−/−^* mice. Genomic DNA from the offspring of the heterozygous crosses were genotyped. The 5′ *loxP* PCR or 3′ *loxP* PCR strategy was used for the genotyping of *Ctdp1^flox/+^* offspring. For the genotyping of *Ctdp1^+/−^* offspring, full-length PCR strategy was used (Fig. 1C). The PCR results can distinguish the genotypes of the mice used in this study (Fig. S1).

### Ctdp1 deletion is embryonic lethal in mice

**Fig. 2.**
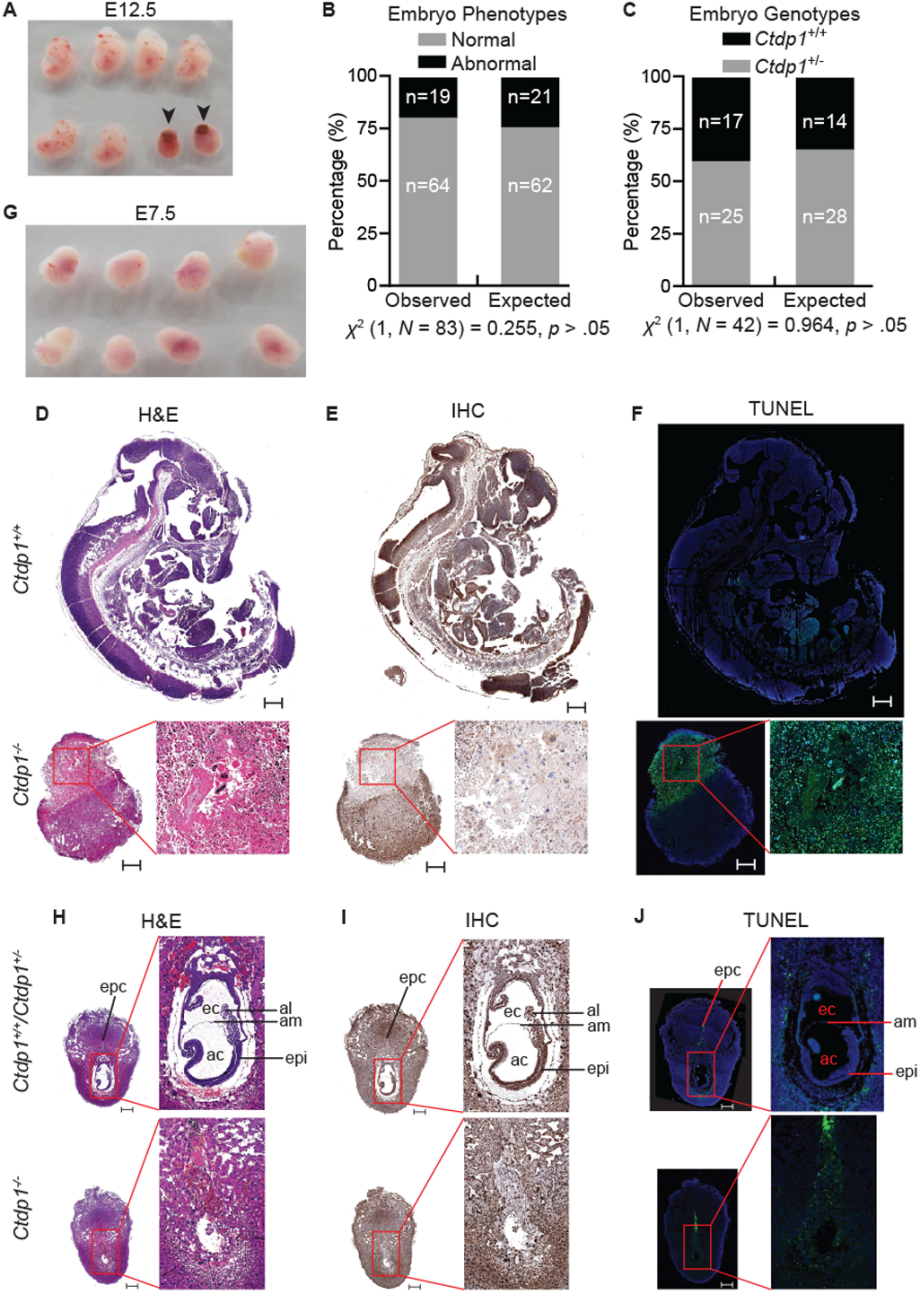
***E2a-Cre* driven *Ctdp1* deletion is embryonic lethal.** (A) Morphology of isolated embryos from *Ctdp1^+/−^* intercrossed mice at E12.5. Abnormal embryos are indicated with black arrows. (B,C) Evaluation of embryo phenotypes (B) and genotypes from normal embryos (C) observed in *Ctdp1^+/−^* intercrosses during E9.5–E16.5. The chi-squared test of independence result is presented below the chart. (D–F) H&E staining (D), Ctdp1 immunohistochemistry (E), and TUNEL staining (F) of *Ctdp1^+/+^* (top) and *Ctdp1^−/−^* (bottom) embryos at E12.5. (G) Dissected maternal decidua from intercrossed *Ctdp1^+/−^* mice at E7.5. (H–J) H&E staining (H), Ctdp1 immunohistochemistry (I), and TUNEL staining (J) of *Ctdp1^+/+^* (top) and *Ctdp1^−/−^* (bottom) embryos at E7.5. Scale bars: 500 µm. The abbreviations in H–J stand for: epc, ectoplacental cone; ec, exocoelomic cavity; ac, amniotic cavity; al, allantoic bud; am, amnion; epi, epiblast.

No *Ctdp1^−/−^* offspring were observed from the *Ctdp1^+/−^* intercrosses after genotyping 103 live born pups, which significantly deviates from expected genotypes (Fig. 1D). Under the hypothesis that *Ctdp1^−/−^* is embryonic lethal, the observed frequencies of *Ctdp1^+/+^* and *Ctdp1^+/−^* genotypes do not significantly differ from expected (Fig. 1E). To determine the approximate time of embryonic lethality in the *Ctdp1^−/−^* genotype, embryos from *Ctdp1^+/−^* intercrosses were evaluated at different stages of gestation. Abnormal (*Ctdp1^−/−^*) embryos could be identified between E9.5–E16.5, which are characterized by a reduction in size and lack of distinguishable developmental features upon gross analysis (Fig. 2A). Assuming biallelic loss of *Ctdp1* disrupts embryogenesis and gives rise to the abnormal embryos within the range of E9.5–E16.5, the numbers of observed normal and abnormal embryos does not significantly differ from the expected frequencies (Fig. 2B). Moreover, within the normal embryos, the frequency of *Ctdp1^+/+^* and *Ctdp1^+/−^* genotypes do not deviate significantly from the expected rates (Fig. 2C).

The E12.5 timepoint was selected to represent the middle stage of embryogenesis (Papaioannou and Behringer, 2012). At E12.5, Hematoxylin and Eosin (H&E) staining indicates the wild-type embryos (Fig. 2D, top) displayed normal development, whereas *Ctdp1^−/−^* embryos displayed characteristics of resorption (Fig. 2D, bottom). Immunohistochemical staining of Ctdp1 in the normal E12.5 embryos (Fig. 2E, top) indicated abundant expression of Ctdp1 in the brain and neural tube, whereas Ctdp1 expression was largely absent in the region containing the abnormal embryos undergoing resorption (Fig. 2E, bottom). The loss of Ctdp1 in the abnormal embryos was accompanied by a high level of cell death indicated by TUNEL staining (Fig. 2F, bottom), whereas a very weak TUNEL staining was observed in wild-type embryo at E12.5 (Fig. 2F, top).

At the earlier timepoint of E7.5, when no morphological differences in decidua are visually apparent (Fig. 2G), H&E staining revealed a lack of normal development in *Ctdp1^−/−^* embryos (Fig. 2H), suggesting that there was a loss of viability in this genotype prior to E7.5. The lack of *Ctdp1^−/−^* embryos in these experiments was not the result of incomplete sectioning (Fig. S2). The *Ctdp1^+/+^* or *Ctdp1^+/−^* embryos have distinct embryonic and extraembryonic regions, but *Ctdp1^−/−^* embryos have no identifiable morphological features expected at this stage of development (Fig. 2H,I). Within the normal developing embryos, Ctdp1 expression is ubiquitous, but most pronounced in the pluripotent epiblast cells (Fig. 2I). The region of the dissected *Ctdp1^−/−^* decidua containing the embryo undergoing resorption was marked by a reduction of Ctdp1 staining (Fig. 2I) and increased cell death (Fig. 2J).

This is the first demonstration that germline deletion of *Ctdp1* in a mammalian model results in embryonic lethality. Here, we observed that *Ctdp1^−/−^* embryonic death occurs by at least E7.5 and is marked by resorption of the embryo and cell death. Further investigations are needed to understand the role of *Ctdp1* in the pluripotent epiblast cells and how this gene supports embryogenesis at the molecular level. Furthermore, it remains to be determined whether loss of Ctdp1 is tolerated in differentiated tissues or if the impacts are specific to stem cells.

### Ctdp1^+/−^ mice are haplosufficient

Western blot analysis determined that the loss of a single *Ctdp1* allele results in a significant reduction of its protein expression in several tissues, including brain, lung, spleen, and heart (Fig. S3A–E). However, *Ctdp1* heterozygosity did not affect animal growth (Fig. S3F) or organ weights (Fig. S3G). Quantitative analysis of complete blood count parameters showed that *Ctdp1^+/−^* mice and *Ctdp1^+/+^* mice were indistinguishable in regards to the numbers and percentages of leukocytes, the number of red blood cells, hemoglobin, and platelets (Fig. S4), indicating no hematologic aberrations in these mice.

One characteristic feature of CCFDN is the hypomyelination of the peripheral nervous system, which results in peripheral neuropathies and impaired motor functions (Kalaydjieva, 2006). However, a battery of behavioral tests determined that monoallelic loss of *Ctdp1* does not significantly affect grip strength, motor function, exploratory behavior, or coordination (Fig. S5A–I). Additional examination of Ctdp1 expression in sciatic nerve determined there was only marginal reduction of its protein levels to approximately 75% of *Ctdp1^+/+^* littermate controls (Fig. S5J–K). It is possible that the threshold to observe CCFDN-like phenotypes could be 30% protein expression as described in CCFDN patients (Varon et al., 2003), whereas in our model system the normal Ctdp1 expression is around 40–50% depending upon the tissue analyzed. The absence of peripheral neurological defects in the heterozygous mice may also be explained by the marginal reduction of Ctdp1 levels in the sciatic nerve compared to *Ctdp1^+/+^* controls or the potential for compensatory function from other HAD superfamily phosphatases. Another possibility is that the specific splice variants caused by the insertion in intron 6 of *CTDP1* found in CCFDN patients could act as a gain-of-function contributing to CCFDN phenotypes.

### Deletion of Ctdp1 leads to cell death in MEFs

**Fig. 3.**
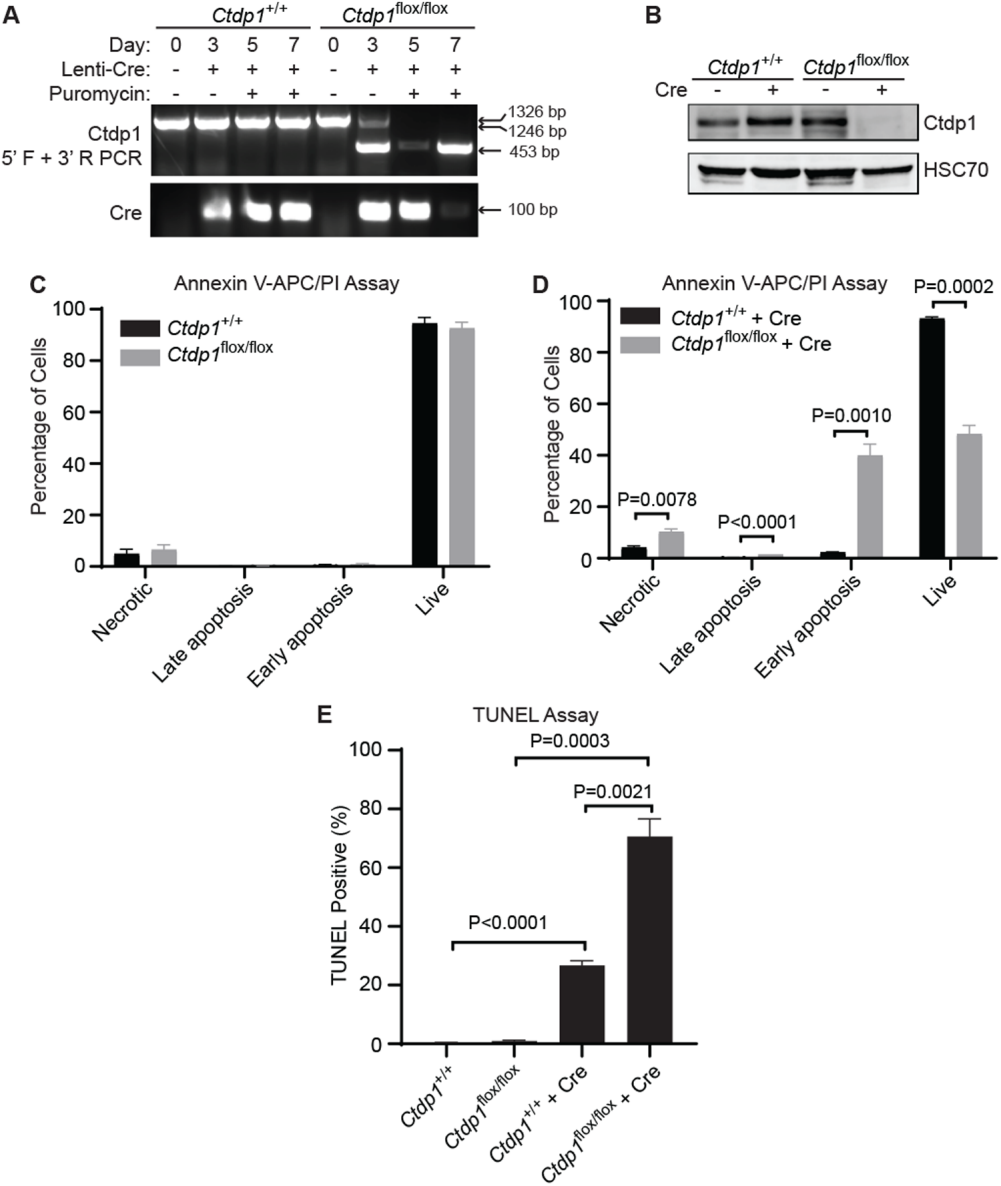
**Ctdp1** **KO** **leads to cell death in MEFs.** (A) *Ctdp1^+/+^* and *Ctdp1^flox/flox^* MEF genotyping at 0, 3, 5, and 7 days post Lenti-Cre transduction. PCR products: floxed allele (1326 bp), wild-type allele (1246 bp), and exons 3–4 deleted (453 bp). (B) Western blot of Ctdp1 in *Ctdp1^+/+^* and *Ctdp1^flox/flox^* MEFs with and without Lenti-Cre transduction and 3 days of puromycin selection. (C,D) Flow cytometry analysis of Annexin V-APC/PI staining of *Ctdp1^+/+^* and *Ctdp1^flox/flox^* MEFs before (C) and 5 days after (D) Lenti-Cre transduction. (E) TUNEL staining in *Ctdp1^+/+^* and *Ctdp1^flox/flox^* MEFs with and without Lenti-Cre transduction. Presented as mean±s.e.m., *n*=3. *P*-values ≤0.05 are displayed.

We generated MEFs as a cell model to study the processes disrupted by loss of *Ctdp1* that may contribute to embryonic death. MEFs were isolated from *Ctdp1^flox/+^* intercrossed embryos at E13.5 and genotyped (Fig. S6A). The isolated MEFs are positive for the fibroblast marker vimentin (Fig. S6B). *Ctdp1* KO was achieved by transducing *Ctdp1^flox/flox^* MEFs with a lentivirus expressing Cre recombinase (Lenti-Cre) (Fig. 3A). Western blot analysis of Ctdp1 confirms that the Cre-mediated deletion of *Ctdp1* results in nearly complete loss of this protein in *Ctdp1^flox/flox^* but not in *Ctdp1^+/+^* MEFs around 3 days post transduction (Fig. 3B).

It was apparent that *Ctdp1^flox/flox^* MEFs suffered from a decrease in viability around 7 days post Lenti-Cre transduction. There was no significant difference between *Ctdp1*^+/+^ and *Ctdp*1^flox/flox^ cell death analyzed by Annexin V/PI staining without Cre expression (Fig. 3C). However, a significant decrease in live cells and concurrent increases in the necrotic, late apoptosis, and early apoptosis populations were observed in *Ctdp1^flox/flox^* MEFs following Lenti-Cre transduction (Fig. 3D). TUNEL staining confirmed that deletion of *Ctdp1* promotes an increase in cell death (Fig. 3E). The loss of viability in *Ctdp1^−/−^* MEFs is consistent with the lack of embryo viability (Fig. 2). These results support the notion that *Ctdp1* is an essential gene necessary for cell viability and indicate that this model could be used to delineate the underlying molecular events associated with embryonic lethality caused by deletion of *Ctdp1*.

### Ctdp1 is essential for cell proliferation and cell cycle regulation in MEFs

**Fig. 4.**
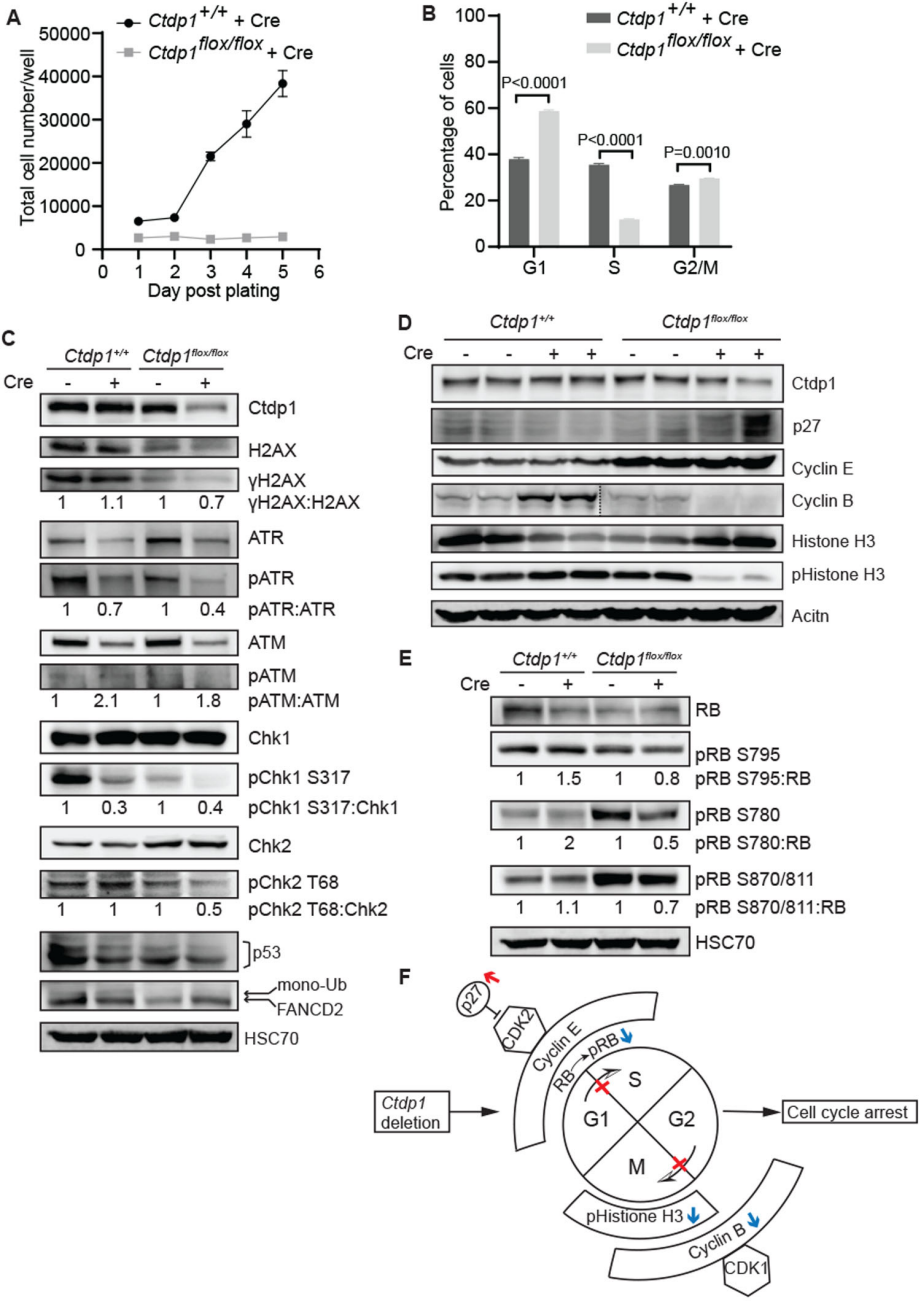
***Ctdp1* deletion impairs MEF proliferation and promotes cell cycle arrest.** (A) *Ctdp1^+/+^* and *Ctdp1^flox/flox^* MEF proliferation after Lenti-Cre transduction. (B) Cell cycle analysis of the *Ctdp1^+/+^* and *Ctdp1^flox/flox^* MEFs 5 days post Lenti-Cre transduction. Presented as mean±s.e.m., *n*=3. *P*-values ≤0.05 are displayed. (C–E) Western blot analysis of DNA damage response proteins (C), G1-S and G2-M transition related proteins (D), and RB phosphorylation (E) in *Ctdp1^+/+^* and *Ctdp1^flox/flox^* MEFs with and without Lenti-Cre transduction. (F) Working model illustrating that *Ctdp1* KO upregulates the CDK inhibitor p27 (red arrow), which functions to inhibit cell cycle progression through G1-S and G2-M transitions. Loss of *Ctdp1* also downregulates (blue arrows) pRB, Cyclin B and pHistone H3 which promote G1-S transition, G2-M transition and M phase entry, respectively.

Elevated transcription activity, which is supported by CTDP1 expression (Juh; z et al., 2012), is commonly seen in highly proliferative cells where it may lessen the rate-limiting constraints on cell proliferation (Lin et al., 2012). This suggests a potential role for Ctdp1 in regulation of proliferation. Indeed, following Lenti-Cre transduction, *Ctdp1*^flox/flox^ MEFs display a significantly slower rate of proliferation when compared with control *Ctdp1*^+/+^ MEFs (Fig. 4A). Indeed, cell cycle analysis indicated deletion of *Ctdp1* in MEFs was accompanied with a significant increase in both the G1 and G2/M phase populations and a decrease in the S phase population compared to *Ctdp1^+/+^* MEFs (Fig. 4B). However, little is known about the regulation of G1 and S phase processes by Ctdp1, which could be attributed to functions of Ctdp1 in the DDR or regulation of cell cycle proteins.

Previous research from our lab discovered that CTDP1 is a novel regulator of the DDR in human breast cells (Hu et al., 2019). Although *Ctdp1*^*flox/flox*^ MEFs exhibit lower expression of total H2AX, there was no induction of its phosphorylated form (γH2AX), a marker of DNA breaks, post Lenti-Cre transduction in either MEF genotype. This could indicate that either DNA lesions are not induced following *Ctdp1* deletion or the upstream kinases are defective. However, the upstream kinases ATR and ATM and their downstream targets Chk1, Chk2, and p53 are only slightly less active in Cre transduced *Ctdp1^flox/flox^* cells compared to *Ctdp1^+/+^* controls (Fig. 4C). Also, there was no changes in the monoubiquitination level of the Ctdp1 interacting protein FANCD2 (Fig. 4C) (Hu et al., 2019), which is involved in ICL repair. The lack of DNA damage and decreases in DDR kinase activation observed when *Ctdp1* is deleted does not explain the observed cell cycle arrest in G1 or G2/M, but are largely consistent with our previous observations in the normal breast tissue derived MCF-10A cells (Hu et al., 2019).

Cell cycle regulators involved in the G1-S and G2-M transitions examined by western blot analysis showed that the cyclin-dependent kinase (CDK) inhibitor p27 was induced by *Ctdp1* KO (Fig. 4D). p27 interacts with a wide range of CDKs and cyclins in diverse stages of cell cycle (Abbastabar et al., 2018). Among them, CDK2, the main target of p27, can complex with Cyclin E or Cyclin A to regulate the G1-S transition (Polyak et al., 1994; Nakayama et al., 1996). Cyclin E is required for the transition from G1 to S phase and Cyclin B/CDK1 regulates progression from G2 to M phase of the cell cycle. We found no alteration in Cyclin E expression level caused by deletion of *Ctdp1* within each cell line. However, Cyclin B expression was decreased in the Cre expressing *Ctdp1^flox/flox^* cells but increased in the *Ctdp1^+/+^* cells under the same conditions (Fig. 4D). Phosphorylation at Ser 10 in Histone H3 (pHistone H3) is an established marker for the onset of mitosis (Crosio et al., 2002). The expression of pHistone H3 in Lenti-Cre transduced *Ctdp1^flox/flox^* MEFs was markedly reduced (Fig. 4D), which also implicates Ctdp1 in the regulation of mitotic entry.

The retinoblastoma tumor suppressor protein, RB, is phosphorylated at multiple sites to promote the release of the E2F1 transcription factor to promote the G1-S transition (Connell-Crowley et al., 1997; Rubin, 2013). Expression of Cre led to a slight increase in pRB species at S795, S780, and S870/811 in the control *Ctdp1^+/+^* cells. However, when *Ctdp1* is deleted, a decrease in pRB is observed (Fig. 4E). The results suggest that loss of *Ctdp1* could prevent the cells from entering S phase by downregulating the RB pathway. Together, these data suggest that *Ctdp1* knockout leads to an increase in p27 and reductions in pRB, pHistone H3 and Cyclin B, which disrupts multiple phases of the cell cycle leading to cell cycle arrest and loss of cell viability (Fig. 4F).

In conclusion, this study has determined that *Ctdp1* is essential for normal mouse embryo development. *Ctdp1^+/−^* is haplosufficient in mice and does not affect growth, hematologic parameters, locomotive function, or manifest CCFDN-like phenotypes. Deletion of *Ctdp1* in MEFs causes cell death, suggesting this effect could prevent normal embryogenesis. *Ctdp1* deletion results in impaired cell proliferation associated with cell cycle arrest and aberrant p27, pRB, pHistone H3, and Cyclin B. While the alteration in these protein expression profiles may be dependent on the role of CTDP1 in transcription, CTDP1 also participates in the post-transcriptional modification of mRNA (Proudfoot et al., 2002; Svetlov and Nudler, 2013). Additionally, CTDP1 temporally controls the cell cycle through regulation of posttranslational phosphorylation modifications of its downstream targets via its protein phosphatase activity (Son and Osmani, 2009; Della Monica et al., 2015; Hégarat et al., 2014; Visconti et al., 2015; Visconti et al., 2012). Thus, it is likely that CTDP1 functions at multiple levels in the regulation of the cell cycle, such as transcriptional, post-transcriptional, and post-translational levels. The mechanisms underlying cell death caused by the loss of *Ctdp1* still need further exploration. The new mouse model developed here provides the prerequisites for future studies to functionally examine the role of *Ctdp1* in cell death, cell cycle, DDR and cancer under controlled physiological conditions. The *Ctdp1^flox/flox^* mice will also facilitate future investigations using cell or tissue-specific conditional KO mouse models to delineate the molecular functions of this gene in cancer and developmental processes.

## MATERIALS AND METHODS

### Animals

All animal maintenance and experimental procedures were in accordance with the National Institute of Health Guide for the Care and Use of Laboratory Animals and approved by the Institutional Animal Care and Use Committee to the University of Nebraska Medical Center. Mice were maintained in a temperature- and humidity-controlled environment with a 12 h/12 h light/dark cycle. Mice had *ad libitum* access to food and water.

### Generation of Ctdp1 KO mice

A conditional floxed allele for *Ctdp1* gene was created by inserting *loxP* sites flanking the exons 3 and 4 by using the *Easi-*CRISPR method described previously (Quadros et al., 2017; Miura et al., 2018) (Fig. S7). Briefly, two synthetic guide RNAs, one each binding to the flanking introns (introns 2–3 and 4–5), were co-injected along with a Cas9 protein and a long single-stranded DNA donor containing the floxed exon cassette into the mouse zygotes derived from C57BL/6 strain. Injected zygotes were transferred into pseudo-pregnant mice, and subsequent steps of mouse genome engineering methods were followed as previously described (Harms et al., 2014). Two sets of genotyping assays, one for each *loxP* insertion, were performed (primers are listed in Fig. S7) and the founder mice containing the correctly targeted alleles were identified and bred to wild-type C57BL/6 mice to establish the mutant lines. Upon Cre mediated recombination the mRNA underwent nonsense mediated decay. This can transpire even if the splicing of the exon 2 with the exon 5 occurs because of the shift in the open reading frame. To generate *Ctdp1* germline KO mice, the floxed mice were crossed with E2a-Cre transgenic mice (Lakso et al., 1996) [B.FVB-Tg(Ella-cre) C5379Lmgd/J] (#003724, The Jackson Laboratory). The resulting heterozygous floxed mice carrying a *Cre* transgene were further intercrossed to produce *Ctdp1* KO mice.

### Embryo dissection and histological and immunohistochemical staining

Timed mating was performed with intercrossed *Ctdp1^flox/+^;E2a-Cre* mice on a C57BL/6 genetic background. Females with copulation plugs were referred as gestation day 0.5. Pregnant females were euthanized at different days of gestation. A tail snip was removed from embryos for genotyping. For embryos at E7.5 of gestation, the embryo and its maternal decidua was isolated together. Embryo specimens were fixed in 10% formalin, dehydrated, embedded in paraffin, and sectioned at 4 µm. For histological analysis, sections were stained H&E according to the standard procedure. For immunohistochemical (IHC) analysis, sections were deparaffinized with xylene, rehydrated with gradually decreasing concentrations of ethanol, and then stained with an CTDP1 antibody (#A301-172A, Bethyl Laboratories, Inc.). The staining procedure followed the manufacturer's instructions. Images of H&E and IHC stained sections were captured with a VENTANA iScan HT Slide Scanner (Roche).

### TUNEL staining

Cell death level in the mouse embryos and MEFs were analyzed by DeadEnd Fluorometric TUNEL system (#G3250, Promega) according to the manufacturer's instructions. Embryos sections were scanned with Cell Discover 7 (Invitrogen). TUNEL stained images were captured by a FLoid Cell Imaging Station (Invitrogen) and more than 100 cells were counted for each sample. Each data point was analyzed with three independent experiments.

### Immunoblotting

Mouse tissue or MEFs were lysed using SDS lysis buffer (50 mM Tris-HCl, pH 6.8, 86 mM β-mercaptoethanol, 2% SDS) supplemented with protease inhibitor cocktail (Sigma-Aldrich) and phosphatase inhibitors (50 mM NaF, 10 mM β-glycerophosphate, 0.1 mM NaVO_4_). Samples were mixed with 5x protein loading buffer and heated at 95°C for 5 min. The protein loading amount used was 80 µg for mice tissue and 25–50 µg for MEFs. Samples were subjected to SDS-PAGE and transferred to a polyvinylidene difluoride (PVDF) membrane. The membrane was blocked in 5% non-fat milk or 5% BSA for 1 h at room temperature and incubated with the primary antibody for overnight at 4°C. The membrane was then washed three times in TBST, incubated with appropriate secondary antibodies conjugated with fluorescent dyes (LI-COR) or HRP (Santa Cruz Biotechnology). All the membranes were visualized with an Odyssey Fc Imaging System (LI-COR). The primary antibodies used for western blots are listed in Table S1.

### Measurement of body weight and organ weights

Animals were fed a regular chow (energy density 3.1 kcal/g) with 19.1% calories from protein, 5.8% calories from fat, and 44.3% calories from carbohydrates (Tedlad diet #7012, ENVIGO). Body weights of mice were measured once per week up to 8 weeks. Mice were euthanized at 8 weeks. The brain, liver, heart, lung, spleen, and kidney from each mouse were collected and immediately weighed. Data were presented as absolute organ weights and their ratios to body weight. The body weight of animals at 8 months of age were also determined.

### Behavioral tests

The sequence of behavioral experiments was an open field test, grip strength test, and rotarod test. Only one type of behavior test was conducted per day, allowing for a minimum of 24 h rest before a subsequent test was performed. Both male and female mice were used for behavioral tests.

### Open field test

The open field test is applied to measure mouse activity and exploratory behavior. Mice were placed in an open 41×41×41 cm box for 30 min and then returned to their home cage. The test for each animal was video recorded with video cameras. Videos were analyzed with EthoVision XT 14 software.

### Grip strength test

Motor strength was measured with a grip strength meter (Columbus Instruments). Mice were positioned to grip a hand bar with both front paws and then gently pulled by the tail from the instrument until they released their grip. The test was repeated five times for each mouse. The average result of each mouse was used for data analysis.

### Rotarod test

The rotarod test was used to evaluate motor coordination and strength and was performed with an accelerating rotarod machine AccuRotor Rota-Rod (Omnitech Electronics, Inc.). The rotation rate increased from 0–40 rpm over 5 min. Four animals were tested concurrently in separate 11 cm wide compartments on a rod approximately 3 cm in diameter and elevated 35 cm. In each trial, the latency to fall from the rod was recorded. The cut-off duration of each trial was 250 s and the average for three trials was evaluated. Each mouse underwent training of three trials per day for three successive days prior to the test day.

### Blood analysis

20 uL of blood was collected from each mouse via maxillary vein and stored in an EDTA coated BD microtainer tube before cell counting. Hematology results were measured by HEMA VET^®^950 analyzer (Drew Scientific Ltd.).

### Generation of stable Ctdp1^+/+^ and Ctdp1^flox/flox^ MEFs

Primary MEFs from mouse embryos were isolated from a female *Ctdp1^flox/+^* mouse bred with a male *Ctdp1^flox/+^* mouse at E13.5 following a previously described protocol (Qiu et al., 2016). PCR was performed for genotype identification. *Ctdp1^+/+^* and *Ctdp1^flox/flox^* MEFs were selected and maintained in a complete medium containing DMEM supplemented with 10% FBS and 1% penicillin-streptomycin (Invitrogen). The primary MEFs were spontaneously immortalized following at least 20 passages.

### Cell culture and lentiviral transduction

293FT cells (Invitrogen) and MEFs were cultured in DMEM medium (Invitrogen) containing 10% FBS and 1% penicillin-streptomycin (Invitrogen) at 37°C in 5% humidified CO_2_ incubators. Lentivirus vector expressing Cre recombinase (Kumar et al., 2008) (#17408, Addgene) was used for deletion of Ctdp1 in *Ctdp1^flox/flox^* MEFs. The transfection of 293FT cells to produce lentiviral particles expressing Cre (Lenti-Cre) was performed using the calcium phosphatase method and ViraPower system (Invitrogen). For the expression of Cre recombinase, MEFs were transduced with lentivirus in the presence of 10 µg/ml Polybrene (Sigma-Aldrich) for 2 days and then either allowed to recover in fresh medium for the indicated time or followed by selection in 1 µg/ml puromycin to remove uninfected cells. Cells were then used further for various tests.

### Cell proliferation assay

Growth curve experiments were performed with indicated cell lines by plating 5000 cells/well in six-well plates. At various time points, cells in individual wells were trypsinized and the total cell number was counted using a hemocytometer. Each data point in the growth curve was analyzed in three independent experiments.

### Annexin V/PI assay

*Ctdp1^+/+^* and *Ctdp1^flox/flox^* MEFs were analyzed for Annexin V-APC (#550475, BD Pharmingen) and propidium iodide (PI) (#P1304MP, Invitrogen) staining according to manufacturer's instructions. Briefly, 5 days post transduction of Lenti-Cre, *Ctdp1^+/+^* and *Ctdp1^flox/flox^* MEFs and their corresponding controls were washed with PBS, trypsinized, and collected in Eppendorf tubes. The cell debris in the supernatant medium was also combined in the same tube. Cell pellets were washed once with cold PBS. After centrifugation at 300× ***g*** for 5 min, the cells were resuspended in 1× Annexin V Binding Buffer (#556454, BD Pharmingen) at a concentration of 1×10^6^ cells per 100 µl. Solutions of Annexin V-APC (5 µl) and PI (2 µl) were added. The samples were incubated for 15 min at room temperature in the dark. 400 µl 1× Annexin V Binding Buffer was added to each tube, followed by data acquisition using an LSR II flow cytometer (BD Biosciences) within 1 h. The results were analyzed using FlowJo software version X.

### Cell cycle analysis

*Ctdp1^+/+^* and *Ctdp1^flox/flox^* MEFs and their corresponding controls were harvested 5 days post transduction of Lenti-Cre. Half a million cells were aliquoted for each sample and fixed in 1 ml of 70% ethanol overnight at −20°C. Next day, cells were centrifuged and washed once with PBS. Then cell pellets were resuspended in 1 ml Telford regent (1 mM EDTA disodium salt, 2.5 U/ml RNAse A, 75 µM PI, 0.1% Triton X-100 in PBS), incubated at 4°C for at least 30 min, and analyzed using a FACSCalibur3 analyzer (BD Biosciences). The percentage of cells within G1, S, and G2 phases of cell cycle were determined via analysis using FACSDiva software version 6.

### Statistical analysis

Data displayed in the figures represent mean±standard error of the mean (s.e.m.) of representative experiments. Statistical comparisons between two groups were evaluated using unpaired two-tailed Student's *t*-test. All numerical data were presented as mean±s.e.m. *P*-values ≤0.05 are considered significant. Data analysis was performed with GraphPad Prism Statistical Software version 8.

## Supplementary Material

Supplementary information
