# Supplementary material for: Ctdp1 deficiency leads to early embryonic lethality in mice and defects in cell cycle progression in MEFs
Source: Biol Open. 2021 Jan 6;10(1):bio057232. doi: 10.1242/bio.057232 (PMC7803460; doi:10.1242/bio.057232)
Supplement: Supplementary information [file biolopen-10-057232-s1.pdf]

Table S1. List of antibodies used in this study.

Name	Vendor	Cat#
CTDP1	Bethyl Laboratories, Inc	A301-172A
HSC70	Santa Cruz Biotechnology	sc-7298
p27	Santa Cruz Biotechnology	Sc-1641
Cyclin E	Santa Cruz Biotechnology	Sc-247
Cyclin B	Santa Cruz Biotechnology	Sc-166210
Histone H3	Cell Signaling Technology	9715
pHistone H3 S10	Cell Signaling Technology	9701
β-actin	Santa Cruz Biotechnology	sc-47778
H2AX	Bethyl Laboratories, Inc	A300-082A
ɣH2AX (pSer139)	Novus Biologicals	NB100-78356
ATR	Cell Signaling Technology	13934
pATR S428	Cell Signaling Technology	2853
ATM	Cell Signaling Technology	2873S
pATM	R&D System	Af1655
ChK1	Cell Signaling Technology	2360
pChk1 S317	Cell Signaling Technology	12302
Chk2	Cell Signaling Technology	2662
pChk2 T68	Cell Signaling Technology	2661
p53	Santa Cruz Biotechnology	Sc-126
FANCD2	Abcam	Ab108928
RB	Cell Signaling Technology	9309
pRB S795	Cell Signaling Technology	9301
pRB S780	Cell Signaling Technology	8180
pRB S870/811	Cell Signaling Technology	8516
Vimentin	Cell Signaling Technology	5741

Biology Open • Supplementary information

Figure S1.

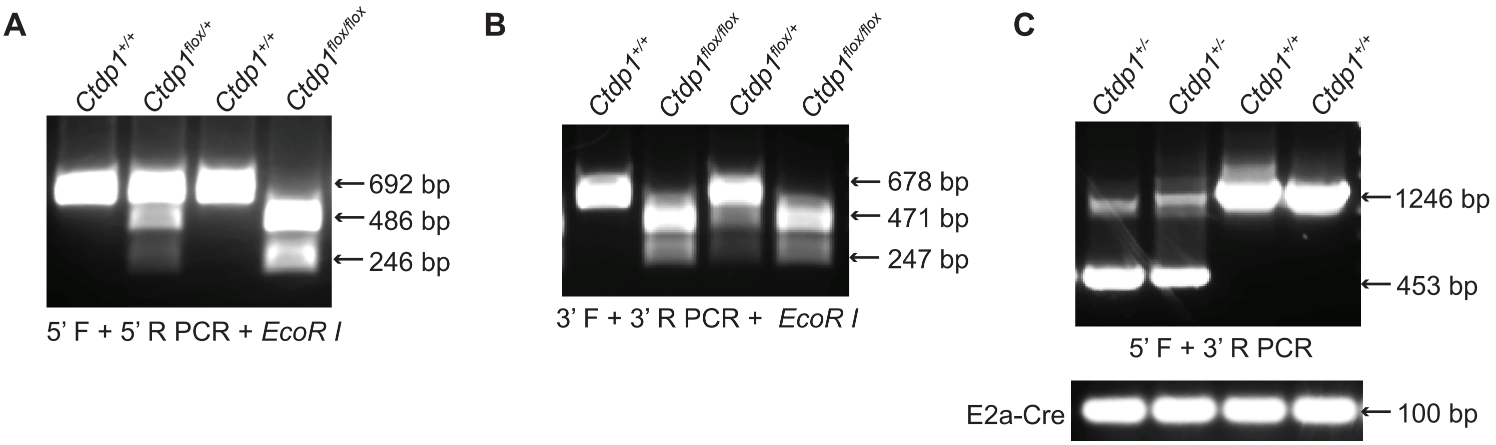

Figure S1. Genotyping PCR reactions

A-B. The representative genotyping results of the *Ctdp1*$^{flox/+}$ heterozygous intercross using 5'F + 5'R primers (**A**) and 3F' + 3R' primers (**B**). The expected sizes of PCR products after *EcoR I* digestion are indicated. *EcoR I* was used here to further distinguish the floxed allele and wild type allele since the size of their PCR products are similar. **C.** The representative genotyping results of the *Ctdp1*$^{flox/+}$*;E2a-Cre* heterozygous intercross using full length 5'F + 3'R primers and E2a-Cre primers, respectively. The expected sizes of PCR products are indicated. The *Ctdp1*$^{-/-}$ genotype is not shown because there are no viable *Ctdp1*$^{-/-}$ pups.

Figure S2.

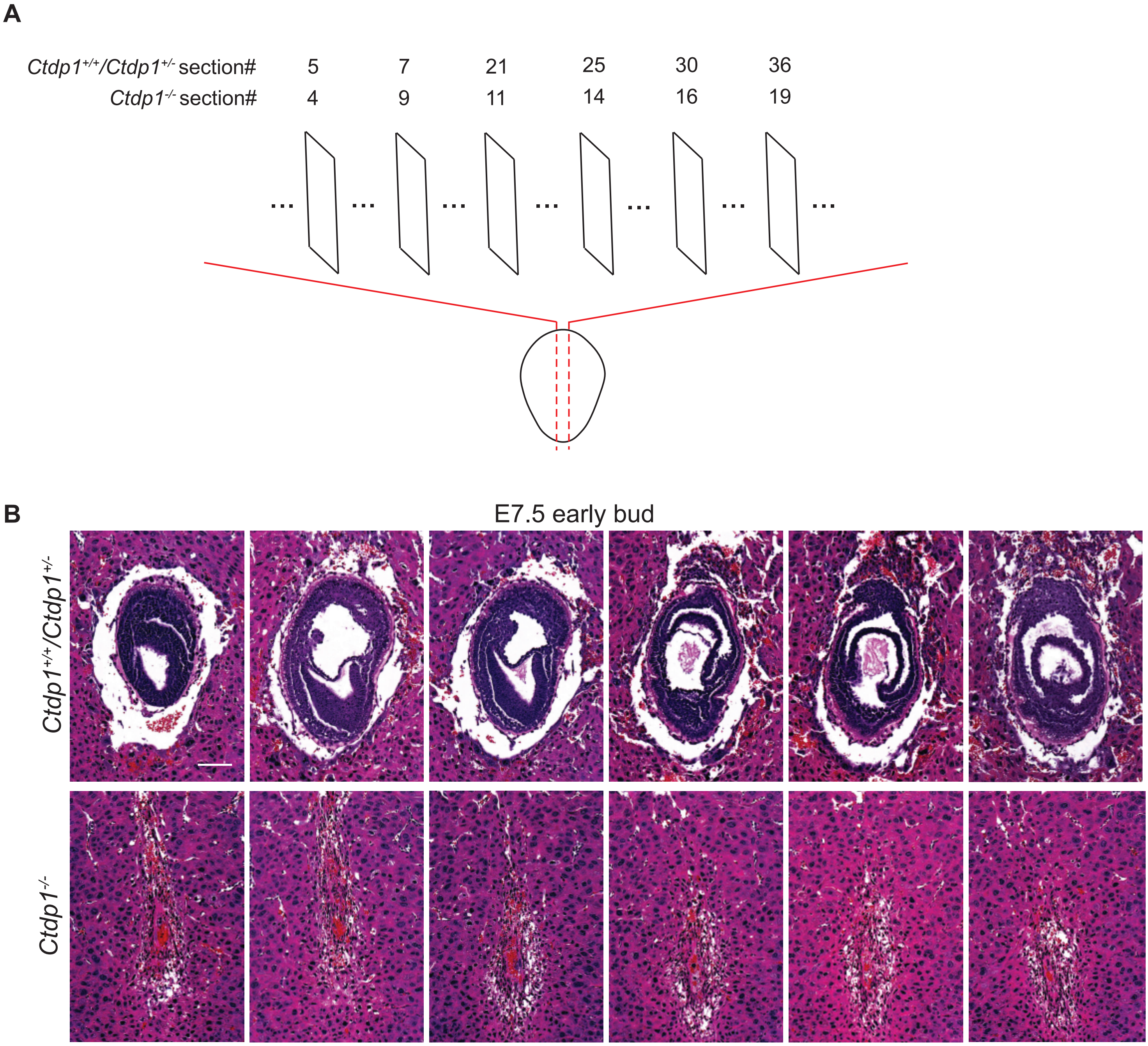

Figure S2. H&E stained serial sagittal sections of representative mouse embryos at E7.5.

A. Schematic showing the serial sections that were evaluated and their relative location within the maternal decidua corresponding to the H&E sections shown in Figure S1B. **B.** Selected representative serial sections of *Ctdp1*$^{+/+}$/*Ctdp1*$^{+/-}$ and *Ctdp1*$^{-/-}$ embryos within the maternal decidua.

Biology Open • Supplementary information

Figure S3.

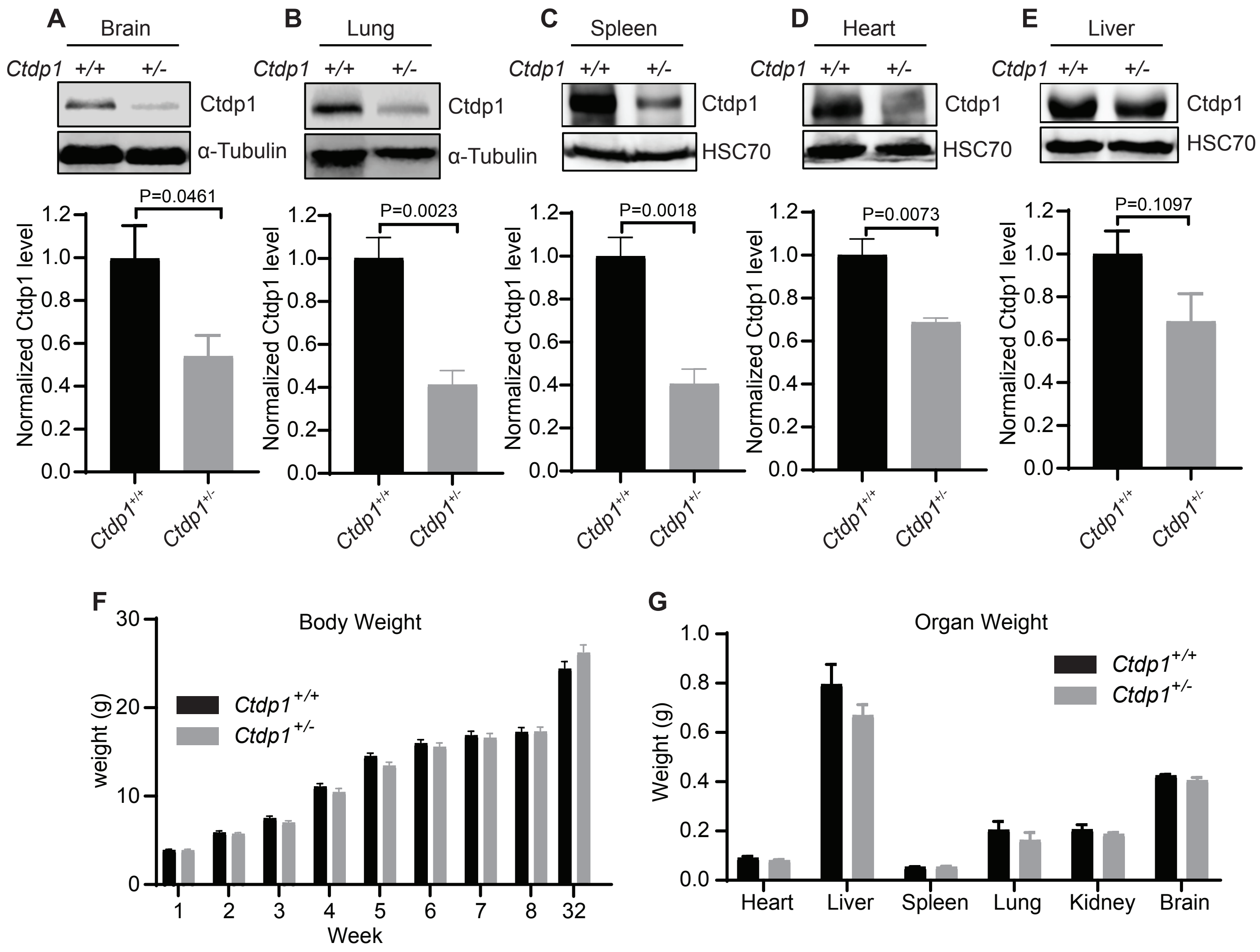

Figure S3. The Ctdp1 expression, body weight and organ weights of *Ctdp1*$^{+/+}$ and *Ctdp1*$^{+/-}$ mice.

A-E. Western blot analysis of Ctdp1 expression of *Ctdp1*$^{+/+}$ and *Ctdp1*$^{+/-}$ mice at 5 weeks in the brain (**A**), lung (**B**), spleen (**C**), heart (**D**), and liver (**E**). Immunoblotting of α-Tubulin or HSC70 was used as a loading control. Normalized Ctdp1 expression was quantified in 4 mice per group depicted in the bar graphs below the protein blots. Values represent the mean ± SEM. Statistical significance was determined using Student's t-test and the P-values of the comparisons are displayed above the bar graphs. **F.** Body weight of *Ctdp1*$^{+/+}$ and *Ctdp1*$^{+/-}$ mice from 1-32 weeks. n = 20 per group for weeks 1-8. n = 4 per group at 32 weeks. **G.** Organ weights of *Ctdp1*$^{+/+}$ and *Ctdp1*$^{+/-}$ mice at 8 weeks. n = 6 per group.

Biology Open • Supplementary information

Figure S4.

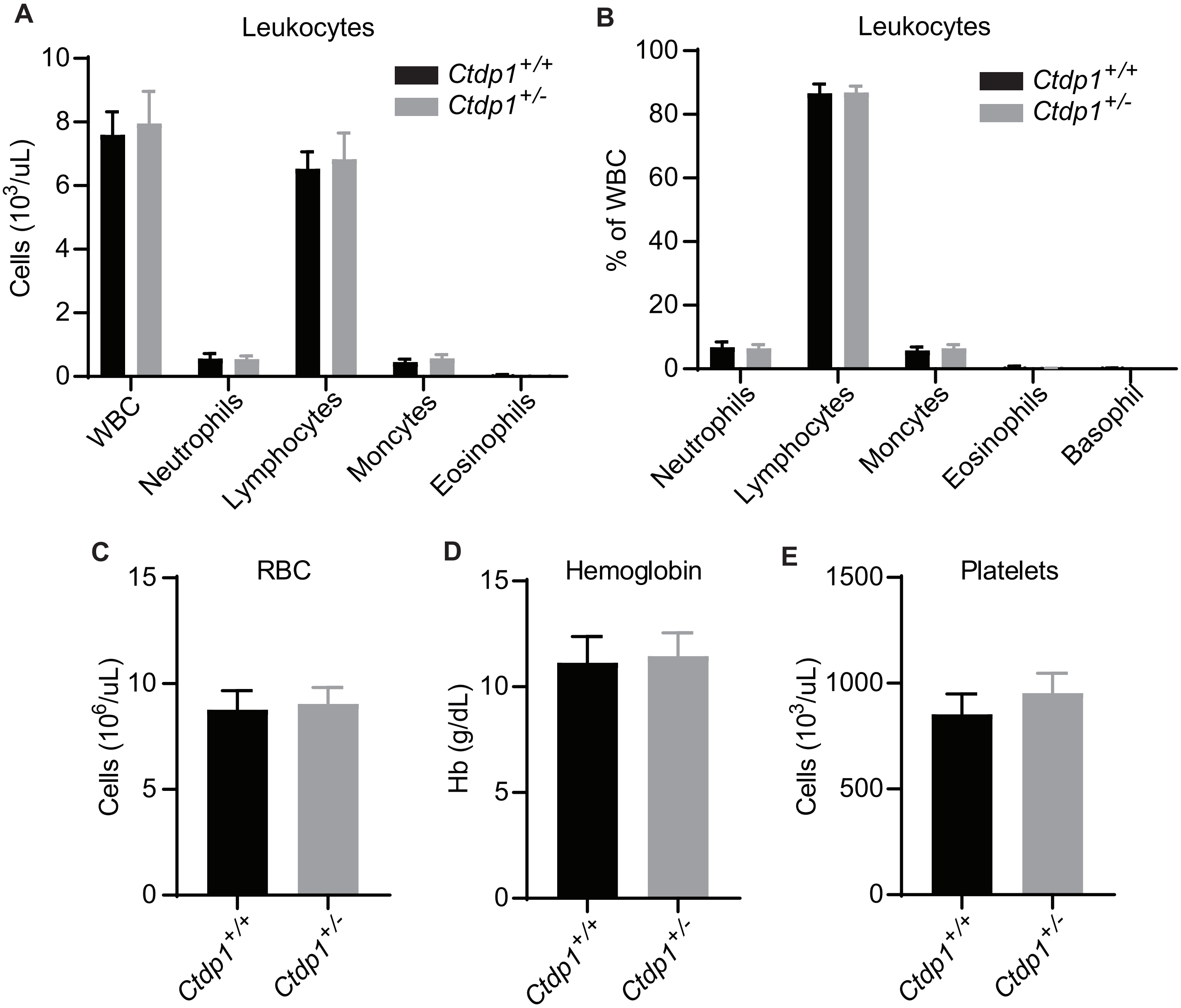

Figure S4. Complete blood evaluation of *Ctdp1*$^{+/+}$ and *Ctdp1*$^{+/-}$ mice.

A-B. The numbers (**A**) and percentages (**B**) of different leukocyte groups in the blood of *Ctdp1*$^{+/+}$ and *Ctdp1*$^{+/-}$ mice at 8 weeks. **C-E.** The number of red blood cells (RBC) (**C**), value of hemoglobin (**D**), and number of platelets (**E**) in the blood of *Ctdp1*$^{+/+}$ and *Ctdp1*$^{+/-}$ mice at 8 weeks. For each of the measurements in panels A-E, n = 6 per group. Data are shown as mean ± SEM.

Biology Open • Supplementary information

Figure S5.

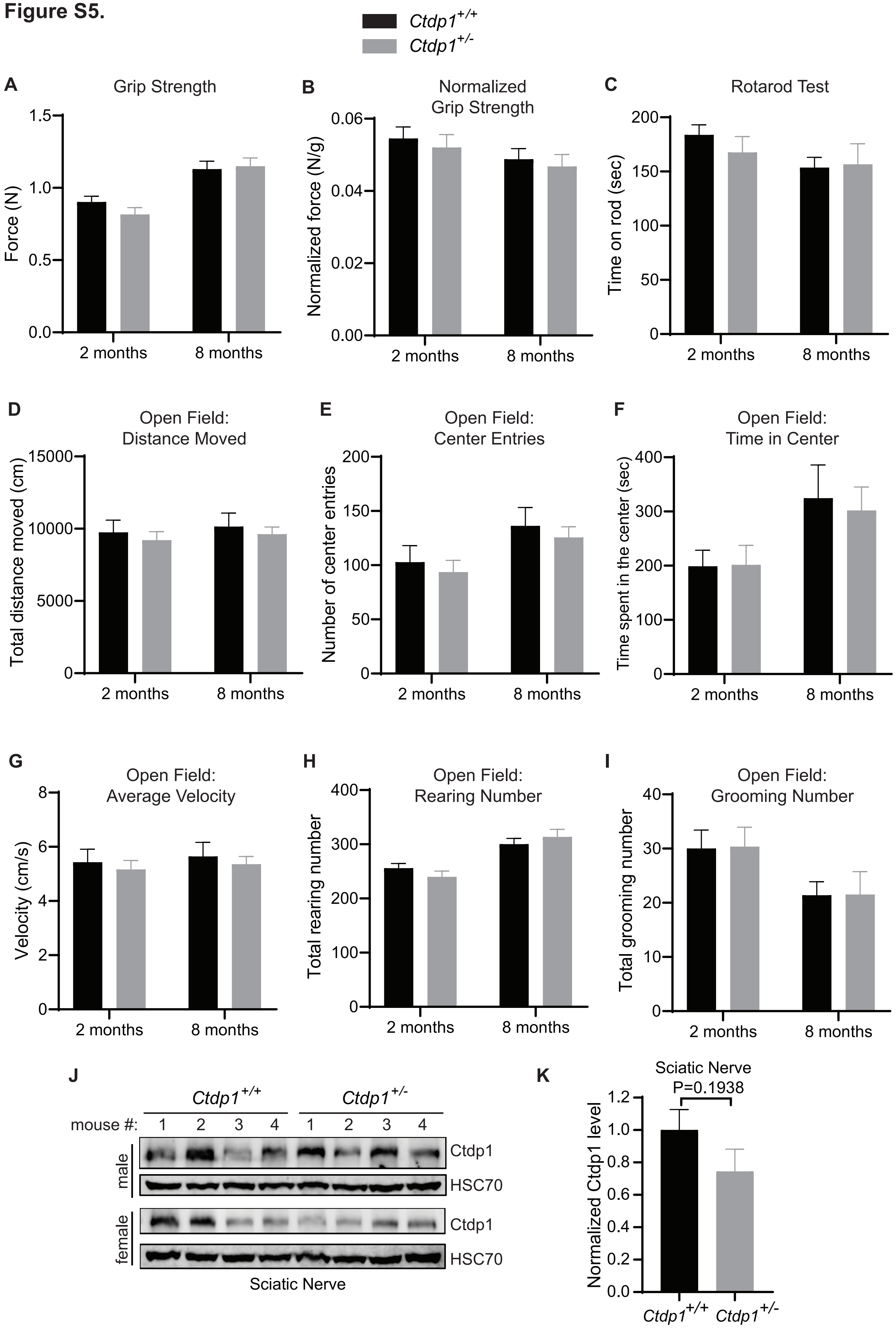

Biology Open • Supplementary information

Figure S5. Locomotive and behavioral tests in *Ctdp1*$^{+/+}$ and *Ctdp1*$^{+/-}$ mice.

A. Measurement of forepaw grip strength in *Ctdp1*$^{+/+}$ and *Ctdp1*$^{+/-}$ mice at 2 and 8 months. **B.** Normalized grip strength value of *Ctdp1*$^{+/+}$ and *Ctdp1*$^{+/-}$ mice at 2 and 8 months. **C.** Measurement of motor function and co-ordination using the rotarod test for *Ctdp1*$^{+/+}$ and *Ctdp1*$^{+/-}$ mice at 2 and 8 months. **D-I.** An open field test was performed at 2 and 8 months to evaluate mouse locomotor activity, including the following parameters: total distance moved (**D**), total number of entries in the center area (**E**), time spent in the center area (**F**), average velocity (**G**), total rearing number (**H**), and total grooming number (**I**). For mice at 2 months, n = 6 per group. For mice at 8 months, n = 8 per group. Data are shown as mean ± SEM. **J.** Western blot analysis of the Ctdp1 protein level in the sciatic nerve of *Ctdp1*$^{+/+}$ and *Ctdp1*$^{+/-}$ mice at 8 months. **K.** Quantitation of the Ctdp1 protein level in the sciatic nerve of the *Ctdp1*$^{+/+}$ and *Ctdp1*$^{+/-}$ mice at 8 months. HSC70 was used as a loading control. n = 8 per group. The P-values are displayed above the bar graphs. Data are shown as mean ± SEM.

Biology Open • Supplementary information

Figure S6.

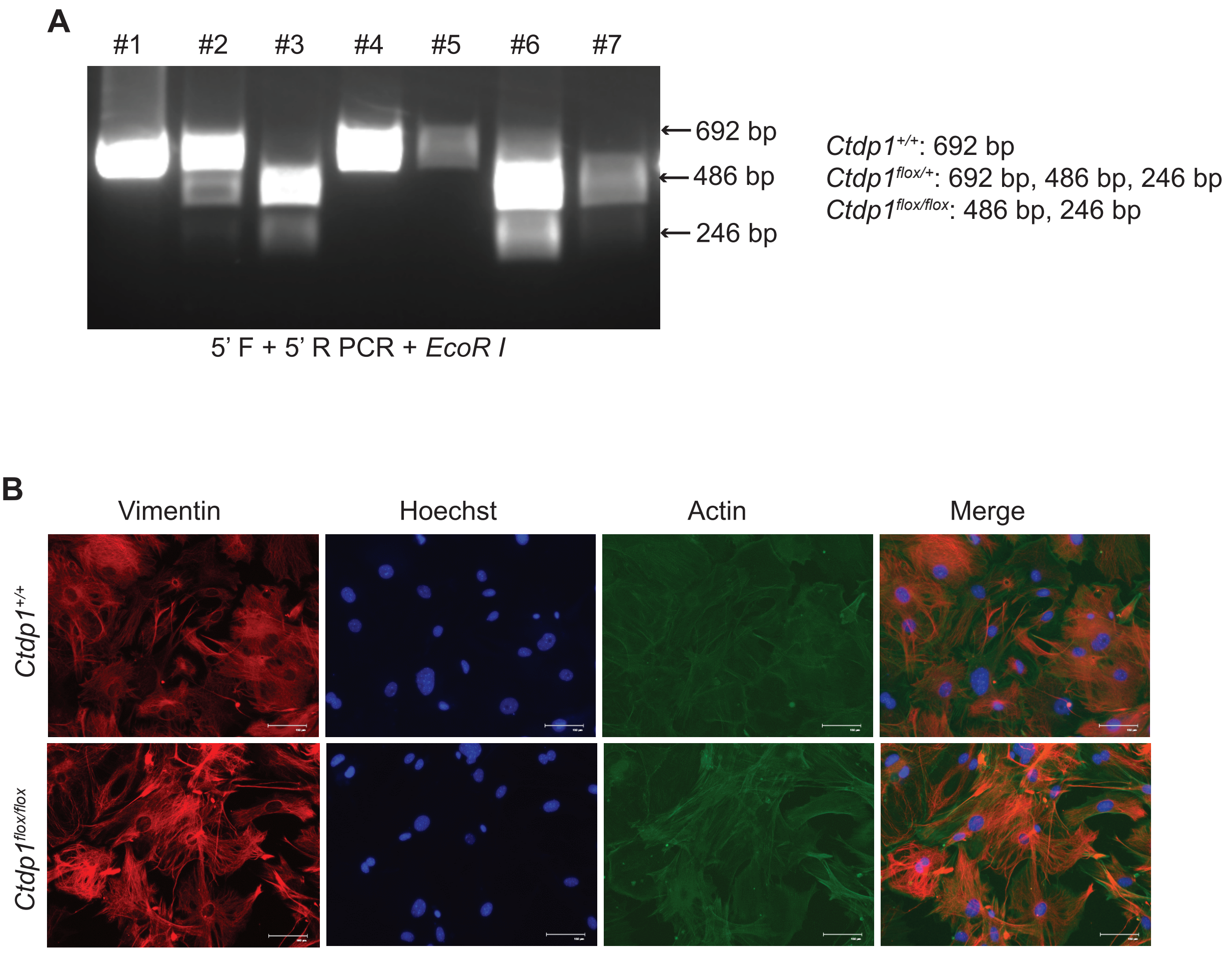

Figure S6. The *Ctdp1*$^{+/+}$ and *Ctdp1*$^{flox/flox}$ MEFs were successfully isolated.

A. Genotyping of the seven embryos derived from a *Ctdp1*$^{flox/+}$ male and a *Ctdp1*$^{flox/+}$ female breeding. #1 and #3 were selected to establish *Ctdp1*$^{+/+}$ and *Ctdp1*$^{flox/flox}$ MEFs, respectively.
B. Immunofluorescence staining of vimentin (fibroblast marker), actin (cytoskeletal marker), and Hoechst (nucleus marker) in *Ctdp1*$^{+/+}$ and *Ctdp1*$^{flox/flox}$ MEFs. Scale bar = 100 µm.

Biology Open • Supplementary information

Figure S7.

REPAIR TEMPLATE with Genomic Sequence

LoxP
EcoRI
Target Exons
5' LoxP forward Primer
5' LoxP reverse Primer
3' LoxP forward Primer
3' LoxP reverse Primer

```
TGCTGGCTTTGCCTTGGCAGCACCTTGAACAGTTTCCCCAGGAAAGAATCCTTTTTGTTTTCCAGCTGTC
TTTCCTCATGCCCTAGATATGTTGAGTATTCTTCTTCTCCAGTATTTCTGGAGGACATAATGCTTTTTGT
CTTCACTGTAAAGACAGCAGATCACCTGCAGTGTTGACAGTTGCCTGCTTTTCTTATGCTCTATGTAGAT
GACCACTGATTCTTAAATGATCTCTATTCATTCTGCAATGTAGGTGGTGGGTGGCTGTTGCTATTTTTTG
TTTGTTTCCATGTAACTGCAGTTGGTTTACTGGGAGAATGGACTCTTGTGCTGACTAGTGACTGCCGTAT
CTGACATAACTTCGTATAGCATACATTATACGAAGTTATGAATTCTGCAGGTATGACCTAGAGCCCAGTT
TCTCATGTTTTCTTTTTTTAAAAGAAAGACAGCATTCACGTAGCTGTCTGTACAGGAGAGTTGTGTGCTA
GGATAGTAGTGTTGGTAACCACTTGCTGAAATCATACCACCACATTCACAGTTATGACAGATGTGCCTTT
CCACACAATGTTAACCAAACCCTTTCCACTCTTACAGGCTGCAGAGTAAAAATGGAAGGCAGCAGGTCCC
CCTTTCCACAGCGACGGTTTCCATGGTGCACAGTGTGCCGGAGTTGATGGTGAGCTCTGAGGTGAGCAGA
GGACTCTGGAGCGTGGATTGATAGGCAGCCCCATCTGAATACTGACTGCTTCCTAAGATGTAGCTCCGCT
GGCTGCCCGTTTCCTTTTTTCCGTTCATAAACAACTGTCTCTCCATGGATGCTTGGAAGCCCCACAGCTC
TTAGTTTTGTAGATTAGTCACCTTCTTGGTACTGCTGACACAGCAGCAGGGGTGGCCTGCATTCCCCTTG
AGCCAGACTTTCCTTAGAAATGCTGTGACAGGCTCCTAACAGGAAGCAGTGCTATGGCTAGGAACTTGTT
TGTGCTCTTTTCTTAATAAAATTGAATTACGTTTTGAATCCAAATATATATACTTTGTCATTTTTCTCAT
TTACTTTTTGACTTTAGCAAGCTGAGAAACTAGGAAGAGAGGATCAGCAGCGACTACATCGAAACAGAAA
ACTGGTTCTCATGGTTGACTTGGACCAGACCCTGATTCATACGACTGAGCAGCACTGTCCCCAGATGTCC
AACAAAGTGAGTGACTCCAGCTTTTCCCTTGGGAATTCATAACTTCGTATAGCATACATTATACGAAGTT
ATTGGCTCTCACAAGAGTGTCCATTTCCAAGTACACTCTCTTGTGTTAAGTGAGTTCCTGGGAACCCTAT
ATGTGGGAGGCAGGTCAGTCAGCTGCTGTGGGCAGACTTCACATAGCTGTGTCTGGGCTGGGCCAGGTGG
CACGGGAAGTGCTCTCTCTCTTCCTGTTGGCATTAGCTTCTGTGTCAGGTTGGCTCACACTTACTAGGGC
CCTTCAAGATAGAAGAGTTCAGAAACAGTCGCTGTGGAAACGGGGACCTGCTGCCTTCCATTTTTACTCT
GCAGCCTATAAGAGTGGAAAGGGTTTGGTTAACACTGCTGGGGGCATGCTCTTGGGAAGAGGTGTGTGTA
GAGTGCCTTAGAGTTAGATGTCTAGAACAAGAGGATATCTGAGGCCATGTT
```

Primers

5' LoxP forward Primer: 5'-GTGACATTTCTGTCGTCTAGTGGA-3'
5' LoxP reverse Primer: 5'-CTAATCAGTGGAAGAACCATGACG-3'
3' LoxP forward Primer: 5'-CAGCCAGCGGAGCTACATCTTAGG-3'
3' LoxP reverse Primer: 5'-CCCTAGTAAGTGTGAGCCAACCTG-3'

Figure S7. Depiction of the *Easi*-CRISPR repair template used to generate the floxed *Ctdp1* allele. The location of the *loxP* sites, targeted exons 3 and 4, *EcoRI* restriction site, and primer sites for PCR genotyping are highlighted.

Biology Open • Supplementary information
